# Development and validation of a machine learning model for clinical wellness visit classification in cats and dogs

**DOI:** 10.3389/fvets.2024.1348162

**Published:** 2024-08-30

**Authors:** Donald Szlosek, Michael Coyne, Julia Riggott, Kevin Knight, D. J. McCrann, Dave Kincaid

**Affiliations:** IDEXX Laboratories, Inc., Westbrook, ME, United States

**Keywords:** wellness visit, machine learning, preventative care, clinical visit, wellness

## Abstract

**Introduction:**

Early disease detection in veterinary care relies on identifying subclinical abnormalities in asymptomatic animals during wellness visits. This study introduces a model designed to distinguish between wellness and other types of veterinary visits.

**Objectives:**

The purpose of this study is to validate the use of a visit classification model compared to manual classification of veterinary visits by three board-certified veterinarians.

**Materials and methods:**

The algorithm was initially trained using a Gradient Boosting Machine model with a dataset of 11,105 clinical visits from 2012 to 2017 involving 655 animals (85.3% dogs and 14.7% cats) across 544 U.S. veterinary practices. Three validators were tasked with classifying 400 visits, including both wellness and other types of visits, selected randomly from the same database used for initial model training, aiming to maintain consistency and relevance between the training and application phases; visit classifications were subsequently categorized into “wellness” or “other” based on majority consensus among validators to assess the model’s performance in identifying wellness visits.

**Results:**

The model demonstrated a specificity of 0.94 (95% CI: 0.91 to 0.96), implying its accuracy in distinguishing non-wellness visits. The model had a sensitivity of 0.86 (95% CI: 0.80 to 0.92), indicating its ability to correctly identify wellness visits as compared to the annotations provided by veterinary experts. The balanced accuracy, calculated as 0.90 (95% CI: 0.87 to 0.93), further confirms the model’s overall effectiveness.

**Clinical significance:**

The model exhibits high specificity and sensitivity, ensuring accurate identification of a high proportion of wellness visits. Overall, this model holds promise for advancing research on preventive care’s role in subclinical disease identification, but prospective studies are needed for validation.

## Introduction

In recent years, artificial intelligence (AI) and machine learning (ML) models have begun to transform veterinary medicine, enhancing diagnostic capabilities and treatment methodologies (1). These technologies are increasingly used to analyze complex data and improve the accuracy of diagnoses across a variety of conditions. For example, machine learning applications in veterinary medicine have shown significant potential in areas ranging from remote sensing in wildlife monitoring to advanced imaging techniques for companion animals (2, 3). Such advancements underscore the growing importance of AI in the field, setting the stage for more specialized applications.

Medical records can provide a wealth of information to support studies of disease prevalence. Within the human medical field, standardized medical coding for insurance and the International Coding of Disease (ICD-11) can facilitate classification of visit types or diagnoses across clinics and hospitals (4). Veterinary medicine lacks a similar classification system that allows for standardized recognition of clinical behavior at visits, and this has been a barrier to studies investigating clinical behavior, results, or outcomes in the field (5). Machine learning techniques are increasingly employed to structure and derive insights from the vast amount of structured, semi-structured, and unstructured data in veterinary medicine, facilitating automated extraction of valuable information from clinical narratives and improving both animal and human health outcomes (1, 6–8).

Wellness or preventive care visits play an important role in the veterinary-client-patient relationship and provide an opportunity to educate clients and identify subclinical disease (9, 10). While companion animal preventative health guidelines to exist, there can be substantial variation between clinics in what is included within a wellness visit and there is no general agreement on what defines a wellness visit or what should be included in wellness care (9–13). Depending on the clinic, a wellness visit could be defined only as the examination of apparently healthy animals with no health concerns, to visits that include preventive care and routine laboratory testing of blood, feces and urine in addition to the physical exam. There is limited information on the results of routine bloodwork—meaning tests ordered without intending diagnosis or monitoring—at various life stages. Most studies that have explored laboratory results in healthy dogs and cats typically have a narrow scope (14–17). They might concentrate on one specific breed, consider only a restricted range of analytes, focus primarily on older pets, or implement a strict definition of what defines “healthy” pets. Currently, large scale real-world evidence (RWE) studies on the value of wellness visits are limited. A major barrier in the collection of large-scale RWE studies is the inability to determine if a clinical examination constitutes a wellness visit due to the non-uniform collection of clinical information.

This study aims to validate a machine learning model designed to classify wellness visits for dogs and cats that have been presented to veterinary practices across the United States. The performance of the model will be benchmarked as compared to the consensus results of three licensed veterinarians who classified a clinical examination as a wellness visit or other visit.

### Model training

The purpose of the visit classification model was to use electronic practice information management records to determine why a pet owner visited their veterinarian (i.e., Why did the pet owner bring their animal to the practice?). A patient visit was defined as when a patient (or patient’s owner) comes to the clinic and obtains one or more products or services on behalf of an animal. The visit begins when the animal walks through the clinic door and ends when the animal leaves the clinic. A patient visit may also be when an animal owner comes to the clinic and obtains some product on behalf of the animal. Each visit was reviewed and classified based on what the intent of the visit was by the pet owner.

Several factors were used to ascertain the intention of a visit including the time since last visit, appointment notes, invoice items, and any available medical notes. The primary sources of information were the medical notes or the stated reasons for the visit. In cases where medical notes were unavailable, transaction details were evaluated. Entries explicitly mentioning ‘yearly wellness exam’ or ‘6-month exam’ were classified as wellness visits. Additionally, if a list of services included routine procedures such as vaccinations, ear swabs, fecal tests, or routine blood work fitting the typical timing of a yearly or six-month checkup, the visit was categorized as wellness. However, wellness visits that coincided with grooming or boarding services were not classified as wellness, since the primary intent was deemed to be grooming or boarding, with medical services provided as a convenience. Visits with unclear intent based on the provided information, or those recorded as mere administrative line items not representing actual activity for the pet, were excluded from our analysis.

During the initial human annotation process, visits were first classified into clinical or non-clinical visit categories. Visits were then classified further as wellness, non-wellness, and non-clinical visits (consisting of boarding, grooming, and retail). Annotators were masked to visit classifications applied by other annotators. Visits were organized by patient at a single clinic. A custom annotation tool was developed to anonymously monitor annotator consensus. It tracked individual annotator metrics, training set agreement, and group agreement on new labels (blind to individual annotators).

A selection of 11,105 clinical visits from 2012 to 2017 was used to train our model. These visits involved 655 animals (85.3% dogs and 14.7% cats) from 544 veterinary establishments in the United States. These visits were randomly selected from the database using the default random number generator (RNG) in base R, the Mersenne-Twister algorithm, to ensure that each visit had an equal chance of being chosen (18). The median duration of a visit was one day (IQR: 1.0–1.0 days) and the median number of transaction line items per visit was two transactions (IQR: 1–5 transactions). We classified the visits into four categories: wellness (24.5%), non-wellness (23.1%), non-clinical (42.5%), and unknown (10.0%). A single visit could have multiple labels with the exception of a non-clinical visit which was defined to be mutually exclusive to all the other categories.

During the model development, to annotate the visits for intent, two methods were used. First, a pair of veterinarians to label each of the 5,984 visits in the preliminary phase (Supplementary Figure S1A). They agreed on 5,058 visits and labeled the remaining 926 as unknown. These unknown visits were excluded from the initial model training. Second, one of the six board-certified veterinarians labeled an additional 5,121 visits. These visits were added to the training dataset along with the agreed-upon 5,058 visits from the preliminary phase.

A comprehensive set of features was used to classify the visits into one of the four categories: wellness, non-wellness, non-clinical and unknown using a Gradient Boosting Machine (GBM) model using the H20 3.20.0.2 and R version 3.5.1 (19, 20). GBM is a decision tree-based algorithm that creates a single model by adding together output from many small, weak decision trees. Each tree is constructed in sequence to fix the errors made by all the previous trees. For a binary classifier (one with 2 classes), the output of the model is the probability that the data provided is in the class (or not in the class). GBM chosen over other methods due to its superior predictive performance and robustness in handling complex non-linear relationships within the data. Furthermore, its ability to provide insights into feature importance and manage diverse data types makes it particularly suitable for our veterinary visit classification task (21). A simple grid search approach was employed to optimize the performance of the Gradient Boosting Machine (GBM). To ensure the validity of the results, the data used for hyperparameter selection did not overlap with any validation or test datasets, thereby avoiding data leakage and ensuring that performance metrics accurately reflect the model’s generalization capabilities. The features used to classify a visit encompassed various demographic and clinical aspects of the visits, including species, age, the number of items on the invoice, total visit cost, days since the last visit, days since the second to last visit, days since the third to last visit, transaction labels, vaccine terms, prescriptions, medical notes (examination type, procedure, etc.), appointment notes words, and appointment reason for visit words. The feature set included all the features available to the veterinarians at the time of annotation. By leveraging this diverse array of features, the model aimed to capture nuanced patterns and relationships in the data, ultimately enhancing its ability to distinguish between wellness visits and other visits in veterinary clinics. The classification model for the classification of wellness visits, was trained employing a 5-fold cross-validation approach, with the F1 score used to select the final trained model. The final GBM model was composed of 111 trees with a maximum depth of 7. The most influential features include vaccine terms, prescriptions, vector born disease test, and visit total cost. Other notable features are laboratory tests medical service, various time-based metrics such as days since the last visit and visit interval, animal age, and several other visit-related metrics. This process resulted in performance metrics including an F1 score of 0.93, a recall of 0.93, a precision of 0.93, and a specificity of 0.97 for the detection of wellness visits as measured on a held-out test dataset of 1,003 visits that was randomly selected from the training set. The held-out set was separate from the training set.

### Training validation annotators

The three veterinarian validators were put through an education period using 125 visits (25 visits from wellness, non-wellness and 75 from the non-clinical visit category) to become familiar with the annotation tool (Supplementary Figure S1B). These visits were selected from the initial 5,058 visits where the label was agreed upon by two veterinarians and were used to train the model (see Model Training Section above). The veterinarians were granted access to the labels that had been collaboratively determined by the two initial veterinarians who categorized the visit. As part of the education process, round table virtual discussions were allowed to gain alignment on classification of visits. These discussions were facilitated by an expert veterinarian involved in the initial training of the model (MC) and the data scientists involved in the development of the model (JR, DM).

After the education period, each of the validators were assessed for agreement to a random selection of 100 visits that matched the distribution of visit types seen in production. The veterinarians were masked to the label of the visits for this assessment. These visits were selected from the initial 5,058 visits where the label was agreed upon by two veterinarians and were used to train the model (see Model Training Section above). A benchmark of 85% agreement (defined *pre-hoc*) to the initial agreed upon label by the two veterinarians used to develop the training dataset was required for the validators to continue to the validation study.

### Development of validation dataset

After the educational period, the three validators were assigned the task of classifying the same 400 visits, distinguishing between wellness visits and other types of visits. To ensure that these 400 visits reflected the distribution of the population of visits expected in a live environment, they were randomly selected from the same database that was used for the initial training of the model, but were not part of the data used to train the model. This was done to ensure consistency and relevance between the training and application phases. To assess the performance of the model for the identification of wellness visits, the results were split into two categories: (1) “wellness” containing the wellness visit type; (2) “other” containing all visits not defined as a wellness visit (for example non-wellness and non-clinical visits). The reference label for each visit was then generated by using the majority consensus for “wellness” or “other” by each of the validators.

### Statistical analysis

A sample size of 400 visits was considered adequate for the identification of wellness visits based off bootstrap simulation using one of the holdout datasets from the k-fold cross validation from the training dataset. A total of 800 visits (double the required amount based on sample size calculation) were collected. In the case that the validators finished their caseload with additional time, they were allowed to continue annotating visits until budget ran out. Consequently, the final validation dataset comprised 622 visits. Initial assessment of validator performance against each other was compared using percent exact match across all three raters and Fleiss Kappa for agreement. The performance of the model in accurately classifying wellness and other visits was evaluated using several metrics, including Sensitivity (Recall), Specificity, Positive Predictive Value (PPV, Precision), Negative Predictive Value (NPV), F1 Score, Balanced Accuracy, Matthews Correlation Coefficient, and Jaccard Index. To address the absence of a gold standard, sensitivity, specificity, and prevalence were estimated using the Expectation–Maximization (EM) algorithm with conditional independence, providing additional measures of the model’s performance (22, 23). To determine the confidence intervals and estimates for these metrics, a bootstrapping approach was applied. Specifically, 2,000 bootstrap samples were generated by randomly sampling visits with the replacement from the original dataset. Bootstrap validation and calibration plots were performed using the Hmisc and rms packages (24, 25). Statistical analysis was done using R version 4.0.2 and various helper functions from the tidyverse (19, 26). Data visualization was generated using ggplot2 (27).

## Results

A total of 622 total visits were classified by all three trained validating veterinarians. Most were dog visits (78.2%, *n* = 487) with 135 cat visits (21.7%). The median age of dogs was 6.0 years (IQR: 2.0–9.3 years) and of cats was 6.0 years (1.7–12.0) with a single cat with no recorded age. A full list of demographic information can be found in Table 1.

**Table 1:** Pet demographic information from 622 veterinary visits.

	Dog visits		Cat visits	
	*n*	%	*n*	%
**Sex**				
Female	33	6.8	6	4.4
Female spayed	200	41.1	55	40.7
Male	44	9	6	4.4
Male neutered	209	42.9	67	49.6
Unknown	1	0.2	1	0.7
**Life stage**				
Juvenile	90	18.5	23	17
Young adult	92	18.9	13	9.6
Mature adult	120	24.6	50	37
Senior	85	17.5	34	25.2
Geriatric	100	20.5	14	10.4
Unknown	–	–	1	0.7

Cat Life Stage: Kitten (<= 1 year), Young Adult (>1 to < = 2 years), Mature adult (> 2 years < = 10 years), senior (> 10 years to < = 15 years), geriatric (> 15 years). Canine Life Stage: Puppy (<= 1 year), Young Adult (>1 to < = 4 years), Mature adult (> 4 years < = 7 years), senior (> 7 years to < = 10 years), geriatric (> 10 years).

### Interobserver agreement

Of the 622 visits, all three trained validators agreed on the classification of 96.9% (602) of the visits with 457 (73.5%) visits being classified as other and 146 (23.5%) being classified as wellness (Fleiss Kappa = 0.91, Supplementary Table S1; Appendix S1). The proportion of visits that have a reference label of wellness is 23.3% (*n* = 145), with 24.8% (*n* = 121) wellness visits classified for dogs and 17.8% (*n* = 24) classified as wellness for cats.

### Model performance

The model demonstrated a specificity of 0.94 (95% CI: 0.91 to 0.96), implying its accuracy in distinguishing non-wellness visits (Tables 2, 3). The model had a sensitivity of 0.86 (95% CI: 0.80 to 0.92), indicating its ability to correctly identify wellness visits as compared to the annotations provided by veterinary experts (Tables 2, 3). This suggests that the model accurately identified 86% of wellness visits. These results indicate that the model accurately recognized 94% of non-wellness and had a low false positive rate. The balanced accuracy, calculated as 0.90 (95% CI: 0.87 to 0.93), further confirms the model’s overall effectiveness (Table 3). In addition, the model was observed to be well calibrated across the predictive range (Supplementary Figure S2). Employing the conditional independence model with the EM algorithm to account for an imperfect gold standard, we obtained a specificity of 0.97 (95% CI: 0.95–0.99) and a sensitivity of 0.85 (95% CI: 0.79–0.90). Additional measures of model performance are found in Table 3 and in Appendix 2.

**Table 2:** Contingency table of model performance to correctly identify wellness visits as compared to reference method (majority of three veterinarians).

		Reference method (3 annotators)		
		Wellness	Other	Total
**Model**	Wellness	20.1% (125)	5.0% (31)	25.1% (156)
	Other	3.2% (20)	71.7% (446)	74.9% (466)
	Total	23.3% (145)	76.7% (477)	100.0% (622)
		Sensitivity	Specificity	
		86.2%	93.5%	

**Table 3:** Bootstrapped model performance of model and 95% confidence interval estimates.

Performance metrics	Estimate	Lower CI	Upper CI
Sensitivity (recall)	0.86	0.80	0.92
Specificity	0.94	0.91	0.96
PPV (precision)	0.80	0.74	0.86
NPV	0.96	0.94	0.97
F1 score	0.83	0.78	0.87
Balanced accuracy	0.90	0.87	0.93
MCC	0.78	0.72	0.83
Jaccard index	0.71	0.64	0.78

F1 represents the harmonic mean of recall and precision. PPV, positive predictive value (precision); NPV, negative predictive value; MCC, Mathew’s correlation coefficient.

## Discussion

Early disease detection in veterinary care depends on the identification of subclinical abnormalities in asymptomatic animals which could be evaluated during a wellness visit (14). Providing a method to determine the type of veterinary visit is essential to determining the benefit of wellness visits. The model developed for this study demonstrated strong specificity and sensitivity, suggesting a robust ability to distinguish between wellness and other visits. The high specificity and sensitivity confirm that the model was able to accurately identify a high proportion of wellness visits. A high specificity in our classification model minimizes the risks associated with incorrectly categorizing other visits as wellness visits. Such a misclassification would have more serious consequences compared to inadvertently classifying a wellness visit as a non-wellness one, as it could potentially lead to overlooking individuals who are in actual need of medical attention or intervention.

This study has limitations that warrant consideration. Primarily, the identification and classification of visit types were conducted using data from specific practice information management systems, which may limit the model’s generalizability across different clinical settings and necessitates additional validation. Furthermore, evaluating visit types is not commonly integrated into the standard veterinary clinical workflow. Therefore, direct comparisons of the model’s capability to accurately identify wellness visits with those documented by a single veterinarian may prove difficult to interpret. To address this, we propose the collection of visit type data at the time of the clinical encounter. Implementing this change would not only provide clearer insights but also potentially eliminate the necessity for using a predictive model, as accurate and structured data capture would be readily available. In addition, the model performance was dependent on the annotations provided by veterinary experts. Our interobserver agreement results, with a Fleiss Kappa of 0.91, underline the consistency among expert annotators, providing a reliable basis for the model’s training. The reliance of the model’s training on the agreement among veterinarians in classifying visits underscores the pivotal role of expert judgment in this process, especially as during the training process the annotating veterinarians could not identify the visit type for 926 visits. It also brings to light the limitations of the model in situations where there is a lack of consensus among experts. To ensure practical relevance, future efforts should aim to integrate this aspect into routine workflow and use more varied data for model training. Additionally, our sample was skewed towards dogs (78.2% of visits), which may have influenced the model’s performance across species. Future research should investigate further refining the model’s ability to differentiate between wellness and non-wellness visits, especially in cases where expert consensus might be challenging. In particular, the discrepancy in wellness visit classification between cats and dogs calls for a more nuanced approach to species-specific care patterns in future model development.

Wellness and preventive care visits are crucial in the veterinary-client-patient relationship, offering a chance to inform clients and detect underlying diseases early. The classification of wellness visits in large-scale RWE data could expand research on the role preventive care plays in the identification of subclinical disease. While the utilization of a visit classification model on retrospective RWE data may expedite the assessment of the clinical value of preventive care visits, further prospective studies are essential to validate these findings.

## Data Availability

The data analyzed in this study is subject to the following licenses/restrictions: the datasets presented in this article are not readily available because the data used in this study contains identifiable transaction and medical history level information. Requests to access these datasets should be directed to donald-szlosek@idexx.com.

## Supplementary material

SUPPLEMENTAL APPENDIX 1 Initial annotator training performance.

SUPPLEMENTARY APPENDIX 2 Model calibration and additional performance metrics.
