# Supplementary material for: Development and validation of a machine learning model for clinical wellness visit classification in cats and dogs
Source: Front Vet Sci. 2024 Aug 30;11:1348162. doi: 10.3389/fvets.2024.1348162 (PMC11392780; doi:10.3389/fvets.2024.1348162)
Supplement: SUPPLEMENTAL APPENDIX 1 — Initial annotator training performance. [file Presentation_1.pdf]

Supplemental Appendix 1. Initial Annotator Training Performance

In this study, the performance of three annotators, referred to as Validator 1, Validator 2, and Validator 3, in labeling wellness and other visits were evaluated. The annotators' match rates were calculated by comparing their annotations to the reference labels that were annotated to train the visit classification algorithm. Validator 1 achieved a match rate of 97.3%, with two instances where visits labeled as "other" were classified as "wellness" according to the reference labels. Validator 2 achieved a match rate of 96.0%, with two "other" visits misclassified as "wellness" and one "wellness visit" misclassified as "other." Validator 3 achieved a match rate of 95.8%, with three "other" visits mistakenly labeled as "wellness." These findings provide insights into the annotators' accuracy and allowed to confidence in moving forward with the validation of the algorithm.

Supplementary Appendix 2. Model Calibration and additional performance metrics.

In our analysis, we used several statistical measures to evaluate prediction accuracy. The Brier Score was 0.074, indicating good probabilistic prediction accuracy. The Harrel's C Index was 0.879, showing strong model discrimination. Calibration curve metrics revealed an index-corrected Emax of 0.008, an index-corrected slope of 0.9686, and an intercept of 0.0073. The near-zero intercept suggests minimal constant or proportion bias across the prediction domain. Overall, these metrics confirm the model's reliability and prediction accuracy.
